# Polymeric Polylactic Acid–Glycolic Acid-Based Nanoparticles Deliver Nintedanib Across the Blood–Brain Barrier to Inhibit Glioblastoma Growth

**DOI:** 10.3390/ijms26020443

**Published:** 2025-01-07

**Authors:** Ying Dang, Zhiwen Zhao, Bo Wang, Aichao Du, Shuangyi Li, Guoqiang Yuan, Yawen Pan

**Affiliations:** 1 The Second Hospital & Clinical Medical School, Lanzhou University, Lanzhou 730030, China; dangy2021@lzu.edu.cn (Y.D.); bwang2020@lzu.edu.cn (B.W.); 220220905611@lzu.edu.cn (A.D.); lshuangyi2024@lzu.edu.cn (S.L.); 2 Department of Neurosurgery, Second Hospital of Lanzhou University, Lanzhou 730030, China; 3 Key Laboratory of Neurology of Gansu Province, Lanzhou University, Lanzhou 730030, China; 4 The College of Chemistry and Chemical Engineering, Lanzhou University, Lanzhou 730030, China; zhaozhw2023@lzu.edu.cn

**Keywords:** GBM, PLGA, BIBF, BBB, nanoparticles (NPs)

## Abstract

The aim of this study was to investigate the inhibitory effect of nintedanib (BIBF) on glioblastoma (GBM) cells and its mechanism of action and to optimize a drug delivery strategy to overcome the limitations posed by the blood–brain barrier (BBB). We analyzed the inhibition of GBM cell lines following BIBF treatment and explored its effect on the autophagy pathway. The cytotoxicity of BIBF was assessed using the CCK-8 assay, and further techniques such as transmission electron microscopy, Western blotting (WB), and flow cytometry were employed to demonstrate that BIBF could block the autophagic pathway by inhibiting the fusion of autophagosomes and lysosomes, ultimately limiting the proliferation of GBM cells. Molecular docking and surface plasmon resonance (SPR) experiments indicated that BIBF specifically binds to the autophagy-associated protein VPS18, interfering with its function and inhibiting the normal progression of autophagy. However, the application of BIBF in GBM therapy is limited due to restricted drug penetration across the BBB. Therefore, this study utilized poly-lactic-co-glycolic acid (PLGA) nanocarriers as a drug delivery system to significantly enhance the delivery efficiency of BIBF in vivo. In vitro cellular experiments and in vivo animal model validation demonstrated that PLGA-BIBF NPs effectively overcame the limitations of the BBB, significantly enhanced the antitumor activity of BIBF, and improved therapeutic efficacy in a GBM BALB/c-Nude model. This study demonstrated that BIBF exerted significant inhibitory effects on GBM cells by binding to VPS18 and inhibiting the autophagy pathway. Combined with the PLGA nanocarrier delivery system, the blood–brain barrier permeability and anti-tumor effect of BIBF were significantly enhanced. Targeting the BIBF-VPS18 pathway and optimizing drug delivery through nanotechnology may represent a new strategy for GBM treatment, providing innovative clinical treatment ideas and a theoretical basis for patients with GBM.

## 1. Introduction

Glioblastoma is a highly malignant brain tumor that arises from astrocytes and is one of the most common malignancies of the central nervous system (CNS) [1]. It accounts for approximately 14.9% of all brain tumors and 47.2% of all primary malignant CNS tumors [2]. Currently, the standard treatment protocol for GBM in adults involves the largest safe surgical resection possible [3], aimed at removing the majority of tumor tissue while preserving as much of the patient’s critical function as possible. Following surgery, treatment typically includes concurrent radiotherapy with temozolomide (TMZ) at a dose of 75 mg/m^2^, with the aim of further prolonging patient survival. Although this combined treatment strategy has improved prognosis to some extent, the median overall survival (MOS) of GBM patients is still less than 15 months, and the 5-year relative survival rate is 35.7% [2]. Most patients face the challenge of tumor recurrence after treatment. With advances in medical technology in recent years, treatments for GBM have evolved to include comprehensive therapies such as surgery, radiotherapy, chemotherapy, immunotherapy, targeted therapy, and supportive care. However, despite the progress made, the overall survival rate for GBM patients remains below 5.5%, and long-term survival is still unsatisfactory [4]. Additionally, the gradual decline in neurological function and the significant reduction in quality of life caused by disease progression have placed an enormous burden on patients themselves, their caregivers, and their families, exacerbating social and economic pressures. In summary, GBM is a highly aggressive malignant brain tumor that poses a major clinical challenge. Although existing treatments can prolong patient survival to some extent, improving long-term survival and quality of life remains an urgent issue that needs to be addressed, and more precise and effective treatment strategies must be explored in the future.

With the continuing development of precision medicine and personalized treatment approaches, the use of numerous small-molecule inhibitors has significantly improved prognosis for patients with a wide range of cancers. Breakthroughs in precision oncology and immunotherapy, in particular, have brought new hope and prospects for tackling aggressive diseases. Meanwhile, the rapid development of nanomedicine in recent years, especially the application of novel NPs in cancer diagnosis and treatment, has led to unprecedented expansion in this field. The unique physicochemical properties of NPs, including high stability, high drug-carrying capacity, the ability to bind both hydrophilic and hydrophobic substances, and high compatibility with multiple drug delivery routes, make them highly attractive for oncology research and clinical applications. NPs can not only efficiently bind, adsorb, and carry small-molecule drugs, but they can also piggyback on a wide range of biomolecules such as DNA, RNA, proteins, and probes, providing innovative ways to diagnose and treat diseases [5]. Particularly in the field of oncology, the existence of natural barriers such as the blood–brain barrier, blood–thymus barrier, gas–blood barrier, and blood–testis barrier often limits the effective delivery of conventional drugs. However, the development of nanomaterials has broken through these limitations and brought new hope for the treatment of complex diseases. In particular, several studies have shown that drug-loaded NPs possess great potential to overcome many challenges faced by conventional drugs [6]. Traditional small-molecule drug therapies encounter issues such as poor efficacy, low bioavailability, and safety concerns, while NPs successfully address these critical problems through encapsulation and targeted drug delivery. The combination of small-molecule drugs with nanomaterials not only improves drug stability and targeting but also reduces systemic toxicity and side effects, thereby enhancing the safety and efficacy of therapy. Additionally, this combination can serve as a precision diagnostic tool, providing potential solutions for the integrated diagnosis and treatment of a wide range of diseases, including tumors, neurodegenerative diseases, and infectious diseases. In summary, the development of small-molecule drugs combined with novel nanomaterials has brought unprecedented benefits to the treatment of cancer patients. This strategy not only accelerates the advancement of precision oncology but also offers new ideas to overcome traditional therapeutic bottlenecks [7]. In the future, as nanotechnology and small-molecule drug design continue to be optimized, novel diagnostic and therapeutic protocols for aggressive diseases such as tumors will progressively achieve greater efficacy, safety, and personalization, bringing new hope for long-term survival and improved quality of life for patients.

Recent studies have shown that nintedanib, an FDA-approved multi-targeted tyrosine kinase inhibitor, affects several key pathways involved in tumor cell survival [8]. These include promoting cell cycle arrest, inducing the endoplasmic reticulum stress response, and modulating glucose transport and autophagy processes [9]. In osteosarcoma cells, nintedanib was found to induce autophagy via the VEGFR2/STAT3/Bcl2 pathway [10]. Additionally, it induces autophagy in lung fibroblasts through a non-traditional Beclin-1-dependent autophagy pathway, effectively alleviating lung fibrosis [11]. In the treatment of malignant pleural mesothelioma, Hegedüs et al. demonstrated that nintedanib could serve as a cytoprotective mechanism to reduce apoptosis independently of the AKT/mTOR and ERK pathways [12]. However, nintedanib still faces significant challenges in treating central nervous system (CNS) disorders. Natural barriers such as the blood–brain barrier (BBB), blood–cerebrospinal fluid barrier (BCSFB), and cerebrospinal fluid–brain barrier severely limit its penetration into the CNS [13], making it difficult for the drug to efficiently reach the tumor site, which in turn affects its targeting effectiveness [14]. Consequently, the specific regulatory pathways of nintedanib in glioblastoma (GBM) therapy and the mechanisms by which it induces autophagy have not been studied in depth, highlighting the urgent need for breakthroughs in this area. To address this issue, polylactic acid–glycolic acid copolymer (PLGA) NPs are considered an ideal drug delivery vehicle and have been FDA-approved for the treatment of cancer, neurological disorders, and hormone-related diseases [15]. Research on PLGA NPs, both domestically and internationally, has focused on improving drug permeability and targeting the blood–brain barrier. PLGA-modified anticancer drugs have emerged as one of the most successful drug delivery systems capable of crossing the BBB. Due to their excellent biocompatibility, controlled-release properties, and degradability, PLGA NPs can effectively deliver drugs to the central nervous system, overcome the blood–brain barrier, and significantly enhance the arrival rate and therapeutic effects of drugs. Based on this, the present study proposes using PLGA NPs as a drug delivery vehicle for nintedanib to facilitate its passage across the blood–brain barrier and accurately target glioblastoma sites, thereby improving the therapeutic efficacy of nintedanib in CNS diseases. Through this approach, we aim to elucidate the specific mechanisms of action of nintedanib on autophagy in GBM cells and develop new therapeutic solutions. The further translation of these findings into clinical applications holds great theoretical value and practical significance, potentially providing new strategies and perspectives for the treatment of aggressive CNS tumors such as glioblastoma.

In this study, we successfully screened BIBF, which exhibits significant GBM inhibitory activity, from a small-molecule drug library using the CCK-8 experimental technique. To better investigate its specific mechanism of action, we comprehensively employed a variety of experimental methods, including transmission electron microscopy observation, plasmid transfection, Western blot analysis, and flow cytometry analysis. Our findings revealed that the mechanism by which BIBF inhibits the proliferation of U251 and U87 cells occurs through the inhibition of the autophagy process. Further studies utilizing advanced techniques such as molecular docking and surface plasmon resonance demonstrated that BIBF specifically binds to the VPS18 protein. This binding interferes with the fusion of autophagosomes and lysosomes in tumor cells, effectively blocking the onset and progression of autophagy and thereby inhibiting tumor cell growth. However, the application of BIBF in GBM therapy is limited by the blood–brain barrier, resulting in a low drug uptake rate when targeting CNS tumors. To overcome this challenge and further improve therapeutic efficacy, we selected PLGA as a drug delivery carrier for BIBF. Validated by in vivo experiments, the results showed that PLGA NPs successfully enhanced BIBF’s ability to cross the blood–brain barrier and significantly improved its therapeutic effect in GBM. In conclusion, the present study systematically elucidated the molecular mechanism by which BIBF inhibits the autophagic process in U251 and U87 cells through its interaction with the VPS18 protein. Meanwhile, the use of PLGA NPs as drug delivery carriers effectively overcome the limitations posed by the blood–brain barrier, significantly enhancing the targeted delivery efficiency and anti-tumor effect of BIBF. This finding provides a strong theoretical basis and practical support for the application of BIBF in the treatment of GBM and opens new research directions for its broader application in other tumors and diseases.

## 2. Results

### 2.1. Effects of Screened Nintedanib on Glioblastoma Cell Biology: Proliferation, Migration, and Invasion

**Figure 1:**
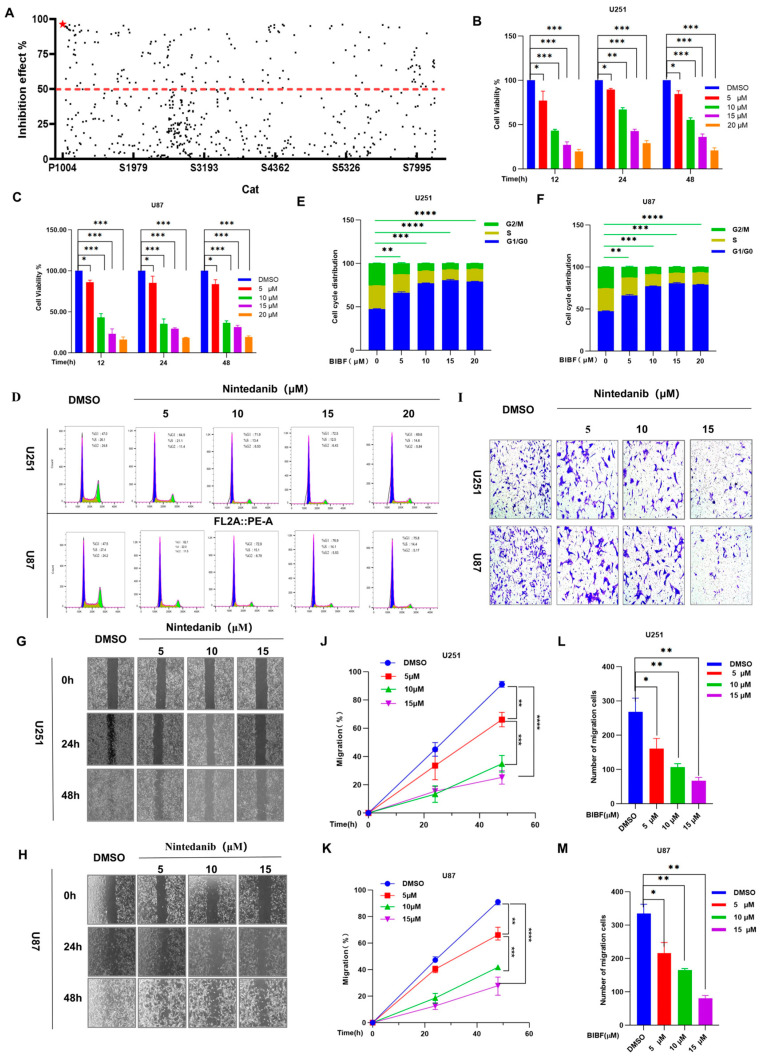
BIBF inhibits the growth of GBM cells. (**A**) Efficiency screening of small-molecule drug library on glioblastoma cells; ★ indicates nintedanib with >95% inhibition. The drugs that achieve 50% cell death are shown above the red dotted line. (**B**,**C**) U251 and U87 cells were treated with different concentrations of BIBF for 12 h, 24 h, and 48 h, respectively. Cell viability was determined by CCK-8 assay. (**D**) U251 and U87 cells were treated with different concentrations of BIBF for 24 h. Inhibition of cell proliferation was detected by flow cytometry. (**E**,**F**) Percentage of cells in different cycles after BIBF treatment of U251 and U87 cells. (**G**,**H**) Cell wound healing ability of U251 and U87 cells treated with different concentrations of BIBF for 24 h. (**J**,**K**) Percentage of U251 and U87 cells that were able to migrate. (**I**) U251 and U87 cells that were treated with the indicated concentrations of BIBF for 24 h. Cells were analyzed for their invasive ability, (**L**,**M**) Number of U251 and U87 cells that permeated through the vesicles, compared to the control * *p* < 0.05, compared to the control ** *p* < 0.01, compared to the control *** *p* < 0.001, and compared to the control **** *p* < 0.0001.

Characterized by low molecular weight, strong targeting ability, good permeability, and ease of chemical modification, small-molecule drug libraries play a central role in the development of new drugs and serve as important tools for antitumor drug screening and development [16]. High-throughput screening (HTS) of these small-molecule drug libraries can rapidly identify candidates with antitumor activity [17], significantly improving the efficiency of drug discovery and development. Additionally, HTS can verify target resistance and elucidate the mechanisms of drug action by interacting with specific tumor targets such as kinases and transcription factors. Furthermore, small-molecule drugs can be utilized to explore non-traditional targets and develop innovative therapeutic strategies, including tumor microenvironment regulation and metabolic inhibition. These approaches address issues related to tumor drug resistance and assist in designing combination drug regimens to enhance efficacy and reduce the incidence of drug resistance. In recent years, advances in chemical synthesis technology and computer-aided drug design (CADD) have continuously improved the size and quality of small-molecule drug libraries [18], facilitating the development of precision medicine and providing crucial support for personalized treatment of tumor patients. In this study, we reviewed the cellular physiological properties and potential side effects associated with the compounds screened, focusing on photosensitizing compounds that significantly induced cytotoxicity and increased reactive oxygen species (ROS) levels. Utilizing a small-molecule drug library, we concentrated on small-molecule compounds with significant inhibitory effects on GBM, with particular attention paid to the tyrosine kinase inhibitor BIBF. The results of our experimental screening demonstrated that BIBF exhibited a very high inhibition rate against GBM (Figure 1A), thus identifying it as a priority drug for further study.

First, we further evaluated the effect of BIBF on the cell viability of U251 and U87 cell lines using the CCK-8 assay. The results showed that cell viability gradually decreased with increasing concentrations of BIBF. However, the changes in the viability of U251 and U87 cells at different time points did not exhibit time-dependent differences (as shown in Figure 1B,C). After treating the U251 and U87 cell lines with various concentrations of BIBF for 24 h, we performed PI staining and analyzed the samples using flow cytometry, which revealed that BIBF significantly inhibited cell proliferation (Figure 1D). Statistical analysis indicated that the inhibition of cell proliferation became increasingly significant (*p* < 0.05) as the concentration of BIBF increased (Figure 1E,F). Additionally, the dose-dependent inhibitory effect of BIBF on the growth of U251 and U87 cells was further validated by cell scratch and invasion assays (Figure 1G–M). Taken together, the results of this part of the experiment suggest that BIBF has potential efficacy in the treatment of GBM and can effectively inhibit the malignant biological behaviors of GBM by influencing multiple mechanisms of cell survival.

### 2.2. Effect of Nintedanib on Apoptosis in Glioblastoma

**Figure 2:**
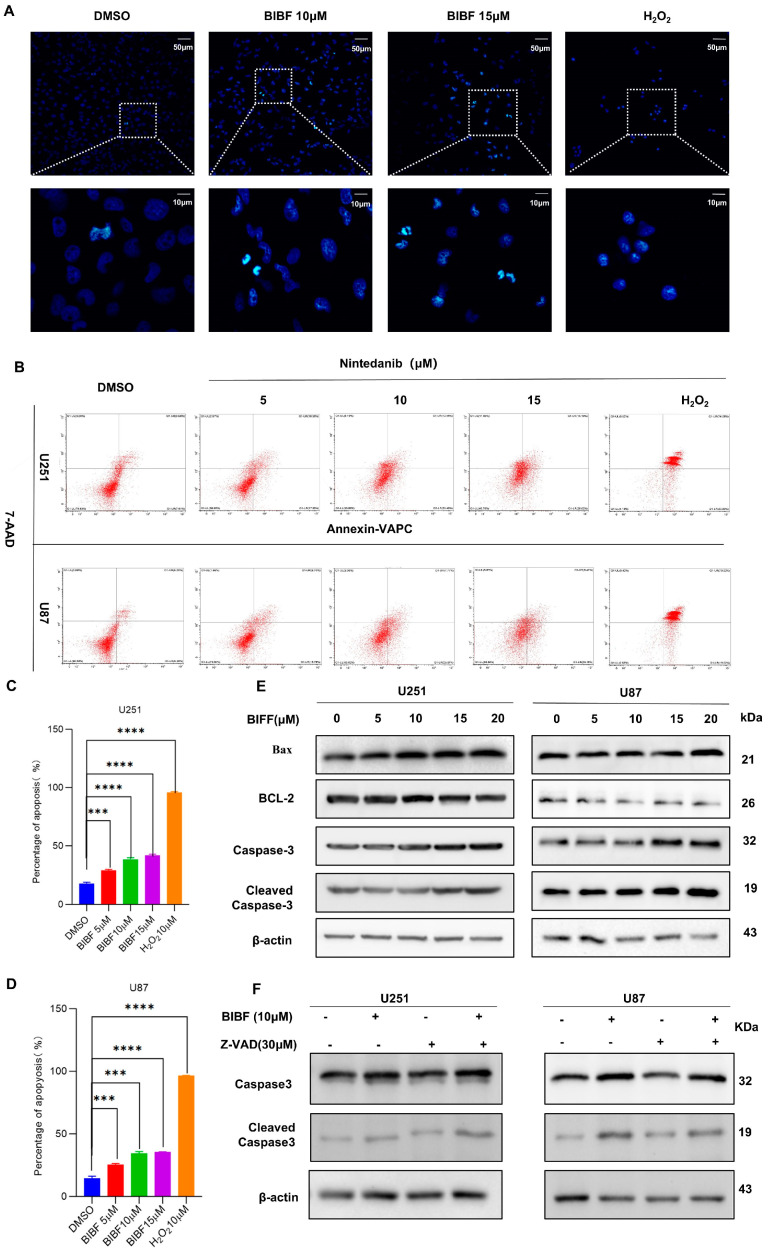
(**A**) U251 cells were stained with TUNEL and DNA breaks were observed by fluorescence microscopy to detect apoptosis. (**B**) U251 and U87 cells were treated with different concentrations of BIBF for 24 h. H_2_O_2_ was used as a positive control, and total apoptosis was detected by flow cytometry. (**C**,**D**) The percentage of U251 and U87 cells undergoing apoptosis is shown in (**B**), compared to the control *** *p* < 0.001, and compared to the control **** *p* < 0.0001. (**E**) U251 and U87 cells were treated with different concentrations of BIBF for 24 h. The relative expression levels of apoptotic proteins Bax, Bcl-2, caspase3, and cleaved-caspase3 were detected by immunoblotting. (**F**) U251 and U87 cells were treated with BIBF (10 μM) or Z-VAD (30 μM) as a negative control for 24 h. The relative expression levels of the apoptosis-related proteins caspase3 and cleaved-caspase3 were determined by immunoblotting.

To investigate whether the inhibitory effect of BIBF on the malignant behavior of glioblastoma cells was mediated by the induction of apoptosis, we designed a series of experiments. First, we treated glioblastoma (GBM) cells with different concentrations of BIBF for 24 h, while a positive control group was established in which glioblastoma cells were treated with 1 mM hydrogen peroxide (H_2_O_2_) for 4 h. As an important reactive oxygen species (ROS), H_2_O_2_ promotes apoptosis under conditions of oxidative stress in the cell [19]. The mechanism of apoptosis induced by H_2_O_2_ mainly involves the induction of oxidative stress, impairment of mitochondrial function, activation of apoptosis-related signaling pathways, and regulation of the expression of apoptosis-related proteins. To detect apoptosis, we used TUNEL staining and observed typical nuclear morphological changes in glioblastoma cells treated with both BIBF and H_2_O_2_. These changes included DNA breaks, chromatin condensation, degradation of the nuclear periplasmic membrane, nuclear blebbing, and other features indicating DNA damage (Figure 2A). Furthermore, to validate BIBF-induced apoptosis, we employed double staining with the membrane-bound protein V and propidium iodide (PI) to quantify the apoptosis rate using flow cytometry. As expected, the apoptosis rate in the BIBF-treated group significantly increased with higher drug concentrations compared to the DMSO-treated control group (Figure 2B–D). These results further indicate that BIBF effectively inhibits glioblastoma growth by inducing apoptosis, providing an important experimental basis for its application in glioblastoma treatment.

Bax is recognized as a key factor in mitochondrial stress-induced apoptosis [20]. It initiates the apoptosis-associated caspase activation pathway by interacting with pore proteins in the mitochondrial membrane, increasing its permeability and leading to the release of cytochrome [21]. Cleaved-caspase-3 is the activated form of caspase-3. As a key executor of apoptosis, it regulates the cleavage of several important proteins either partially or completely, and its expression is closely related to apoptosis [22]. To verify the effect of BIBF on apoptosis in glioblastoma cells, we analyzed the expression levels of Bax, Bcl-2, caspase-3, and cleaved-caspase-3 using protein blotting. The results showed a significant decrease in the expression of the anti-apoptotic protein Bcl-2 and a significant increase in the expression of the pro-apoptotic protein Bax with increasing concentrations of BIBF. Additionally, the expression levels of both caspase-3 and cleaved-caspase-3 were also significantly elevated (Figure 2E). This outcome was consistent with the flow cytometry data, further supporting the role of BIBF in the regulation of apoptosis.

Z-VAD is a widely used apoptosis inhibitor in apoptosis research [23], primarily functioning to inhibit the activity of caspase family proteins, thereby blocking the execution phase of apoptosis. It effectively suppresses the activity of several caspase family members and prevents the activation of key apoptotic signaling pathways, particularly those mediated by the mitochondrial and death receptor pathways. This makes Z-VAD an important tool for researchers exploring the mechanisms of apoptosis, developing anti-apoptotic drugs, and studying the cell death processes in disease models. In this study, we applied Z-VAD as a negative control to U251 and U87 cells and analyzed the changes in apoptosis using Western blot (WB) analysis. The results showed that Z-VAD was able to reverse the BIBF-induced increase in cleaved caspase-3 expression levels when cells were treated with both Z-VAD and BIBF (see Figure 2F). In conclusion, BIBF can induce apoptosis in GBM cells and thereby inhibit the malignant progression of GBM.

### 2.3. Inhibition of Glioblastoma Autophagy by Nintedanib

**Figure 3:**
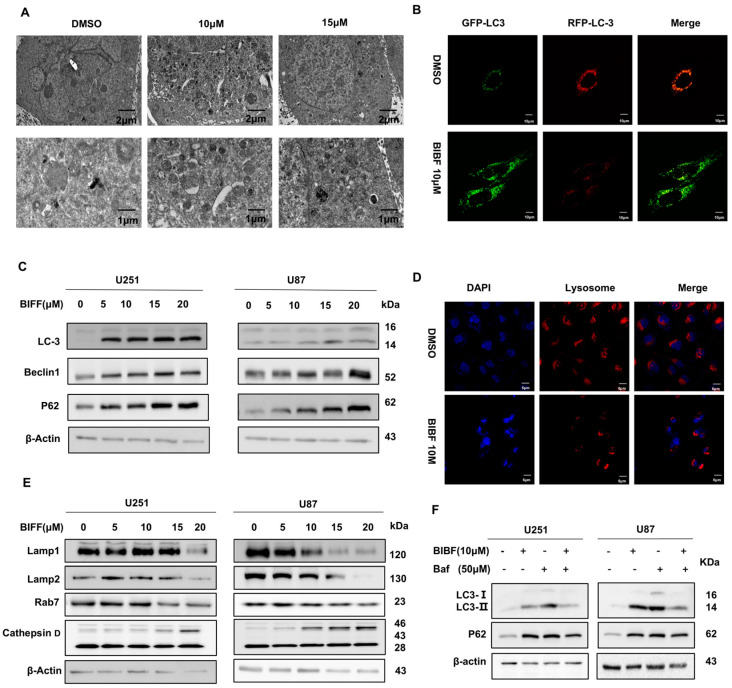
BIBF inhibits autophagy in GBM. (**A**) After BIBF treatment of U251, apoptotic vesicles (black color) and autophagic vesicles (monolayer vesicle structure) were observed via transmission electron microscopy. (**B**) Cells were transfected with plasmid encoding GFP-RFP-LC 3, and the number of yellow spots and red spots of autophagic vesicles fused with lysosomes was reduced compared with control when BIBF acted on U251 cells for 24 h as observed by confocal microscopy. (**C**) U251 and U87 cells were treated with different concentrations of BIBF for 24 h. The relative expression levels of autophagy proteins LC-3, Beclin1, and P62 were detected by immunoblotting. (**D**) After BIBF treatment of U251, lysosomal probes were used to label lysosomes in live cells, which showed altered lysosomal morphology compared to the DMSO group. (**E**) Lysosomes from U251 and U87 cells treated with different concentrations of BIBF for 24 h were analyzed by immunoblotting assay to determine the relative expression levels of Lamp1, Lamp2, Rab7, and Cathepsin D. The lysosomes were labeled with a lysosomal probe. (**F**) Analysis of immunoblotting to measure the relative expression levels of autophagy-related proteins LC-3 and P62 by treating U251 and U87 cells with BIBF (10 μM) alone or with Baf (50 μM) for 24 h.

Apoptosis and autophagy are regulated by several common upstream signals and can cross-regulate each other [24]. Apoptosis is the orderly degradation and removal of cells and represents an active process of cell death [22]. Autophagy, on the other hand, is a cellular self-degradation process that involves the formation of autophagosome [25]. These autophagosomes encapsulate intracellular damage and excess components, subsequently fusing with lysosomes for degradation. Although apoptosis and autophagy are functionally distinct, their interactions play a crucial role in cellular physiology and pathology [24]. To investigate whether the effect of BIBF on apoptosis in GBM cells is related to autophagy, we first examined BIBF-treated GBM cells using electron microscopy and found that the number of autophagic vesicles was significantly increased compared to the control group (Figure 3A). To further verify the effect of BIBF on autophagic flux in GBM cells, we transfected the GFP-LC3-RFP plasmid into U251 cells. Upon autophagy induction, we observed an increase in the number of yellow and red spots. When autophagy was inhibited, the percentage of yellow spots was significantly higher, indicating that fusion between autophagosomes and lysosomes was reduced, resulting in a decrease in both yellow and red spots due to degradation within the autophagic lysosome. Our experimental results suggest that BIBF may inhibit the autophagic process in GBM cells (Figure 3B). The presence of LC3 in autophagosomes and its conversion to the downward-migrating LC3-II form are hallmarks of autophagy [26]. Beclin-1 is a key regulatory protein involved in the autophagy-lysosomal degradation process [27], while P62 plays a role in the degradation of proteasomes and the triggering of lysosomal proteins [28], a reduction in its level is positively correlated with autophagic activity. We analyzed the expression of these proteins using Western blotting (Figure 3C). The results showed that the expression level of LC3 protein increased in a dose-dependent manner, and the expression level of Beclin-1 protein also increased in a dose-dependent manner, while the expression level of p62 protein decreased as the concentration of BIBF increased. These findings suggest that BIBF does indeed inhibit the autophagic process in GBM.

The effects of BIBF on autophagy and lysosomal function in GBM cells are manifested through changes in several molecules and their biological processes. Studies have shown that LAMP1 and LAMP2 are major transmembrane glycoproteins of the lysosomal membrane involved in lysosome–phagosome fusion and cholesterol transport [29]. Their decreased expression levels affect the structural stability and function of lysosomes. Additionally, Rab7 is localized to late endosomes and primarily regulates the maturation of autophagosomes and their fusion with lysosomes [30]. The reduction in its expression level suggests that nintedanib may interfere with the membrane transport and fusion of autophagosomes and lysosomes by inhibiting Rab7, which in turn inhibits the autophagy process. Cathepsin D is involved in normal protein degradation [31]. During apoptosis, cathepsin D is secreted into the cytoplasm and participates in the cleavage of substrates in the apoptotic pathway [32]. It was found that the morphology of lysosomes was significantly altered after 24 h of BIBF treatment of glioblastoma cells, as indicated by lysosome–specific probe labeling. Meanwhile, Western blot analysis showed that the expression levels of LAMP1 and LAMP2 proteins were significantly decreased in a dose-dependent manner, suggesting that the integrity and function of lysosomal membranes may be impaired. The expression of the Rab7 protein also decreased with increasing concentrations of BIBF, further suggesting that BIBF may inhibit autophagy by affecting membrane transport and fusion. Furthermore, the level of the cathepsin D precursor protein increased with higher concentrations of BIBF, which may represent a stress response triggered by cellular stimulation from BIBF aimed at removing intracellular harmful substances by enhancing the degradation capacity of lysosomes. In conclusion, BIBF inhibits autophagy and promotes the accumulation of toxic substances in glioblastoma, leading to the onset of apoptosis by decreasing the expression levels of LAMP1, LAMP2, and Rab7 while impairing lysosomal function and its ability to fuse with autophagosomes.

To further confirm the inhibitory effect of BIBF on autophagy in GBM cells, we used the autophagy inhibitor bafilomycin (Baf) as a positive control. Baf is a classical autophagy inhibitor that prevents lysosomal acidification by inhibiting V-ATPase activity, thereby interfering with the fusion of autophagosomes with lysosomes and blocking the final stage of autophagy. In our study, U251 and U87 cells were treated with BIBF (10 µM) and Baf (50 µM), either alone or in combination. The experimental results showed that the expression level of LC3B, an autophagosome marker, was significantly decreased, while the expression level of p62, an autophagy substrate, was significantly increased when BIBF and Baf were combined, as detected by Western blot (Figure 3E). This phenomenon suggests that the inhibition of autophagy by Baf was further enhanced by the addition of BIBF. Additionally, when BIBF or Baf was used alone, the expression level of p62 was also significantly higher than that of the control group (Figure 3F), further confirming that BIBF can inhibit the autophagic process in GBM cells.

### 2.4. Nintedanib-Mediated VPS18 Pathway Affects Autophagy

**Figure 4:**
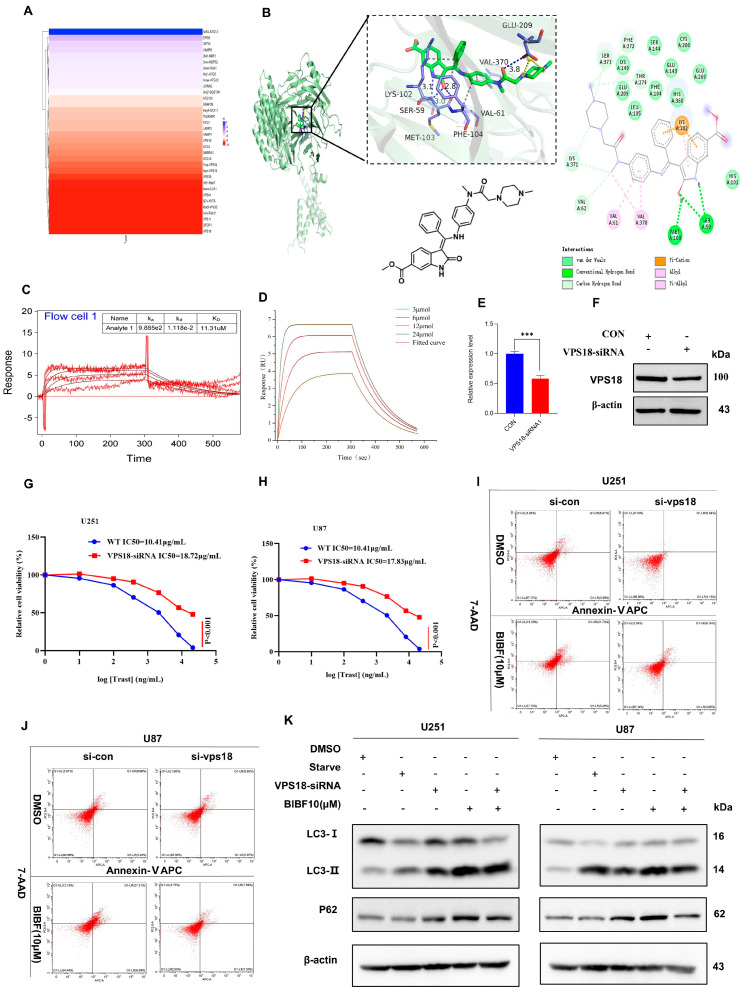
BIBF is impairing autophagy by affecting VPS18. (**A**) Heat map of the docking force of BIBF with autophagy molecules. (**B**) Details of the binding interaction between BIBF and VPS18 are shown. (**C**) Kinetic constants (Kd) of the interaction between BIBF and VPS18 were analyzed using surface plasmon resonance (SPR), “e” represents “multiplied by 10 to the power of”. (**D**) SPR kinetic analysis showed that the Kd value of BIBF to VPS18 was 1.118. (**E**) qPCR verified the VPS18-siRNA knockdown effect, compared to the control *** *p* < 0.001. (**F**) WB verified the VPS18-siRNA knockdown effect. (**G**,**H**) Viability assay of glioblastoma by BIBF after VPS18 knockdown. (**I**,**J**) Effect of BIBF on glioblastoma apoptosis after VPS18 knockdown was analyzed by flow. (**K**) Effect of BIBF on glioblastoma autophagy after VPS18 knockdown analyzed by WB.

Molecular docking technology predicts the binding mode and binding strength between drug and target molecules by computer simulation, which can provide three-dimensional structural information of drug–target molecule interactions [33]. Combined with molecular dynamics simulation, it helps to reveal the structure–function relationship between the drug and the target. Molecular docking technology is important for autophagy regulator discovery, intermolecular mechanism of action analysis, targeted drug design, and precision medicine research [34]. In our study, the binding mode and binding strength between BIBF and autophagy-related molecules were investigated by molecular docking technology, and the binding ability was evaluated by scoring values: the range of scores for the binding score illustrates that a scoring value of less than −7.0 indicates that the target has a strong binding ability to the compound; a scoring value between −5.0 and −7.0 indicates a better binding ability; and a scoring value between −5.0 and −4.25 indicates a better binding ability. A scoring value between 5.0 and −4.25 indicates the presence of some binding ability [35]. The simulation results showed that BIBF was able to effectively bind to the active pocket of VPS18 with a Glide-Docking score of −9.9 kcal/mol (much less than −7.0), indicating that BIBF has a strong binding ability to VPS18. By analyzing the three-dimensional interactions, it was further revealed that the molecular mechanism of BIBF binding to VPS18 was the hydrogen bonding interactions formed by BIBF with the amino acid sites of SER59, MET103, PHE104, and VAL370 of VPS18, with hydrogen bonding distances of 3.1, 3.0, 2.8, and 3.8, the hydrophobic interactions formed by BIBF with the amino acid sites of LYS102 and VAL61 amino acid sites formed hydrophobic interactions; BIBF formed a salt bridge interaction with GLU209 of VPS18. The above interactions stabilized the binding of BIBF to the active pocket of VPS18, forming a complex (Figure 4B). In conclusion, the binding of BIBF to VPS18 is mainly dependent on hydrogen bonding, hydrophobic interactions, and salt bridge interactions. The binding scores indicate that BIBF binds well to the active pocket of VPS18, and the binding mode has high stability.

To further validate the interaction between BIBF and VPS18 proteins in molecular docking assays, we used the surface plasmon resonance (SPR) technique to experimentally validate the interaction between BIBF and VPS18 using the Biacore instrument. SPR is a real-time, label-free detection technique based on optical physics principles for the study of molecular interactions, particularly the binding of drugs to active molecules [33]. This technique not only provides experimental validation of molecular docking calculations but also facilitates the clinical translation of drug design. Through SPR, interactions such as hydrogen bonding, hydrophobic interactions, and salt bridges predicted in molecular docking can be verified to be real, thus optimizing the molecular docking model. In addition, SPR can provide quantitative parameters of drug–target binding. While traditional molecular docking results usually measure the binding ability in terms of binding energy, SPR can provide more accurate kinetic parameters, such as equilibrium dissociation constant (KD), reflecting the affinity between the drug and the target molecule, and binding rate constant (kon) and dissociation rate constant (koff): reflecting the rate and stability of molecular binding, respectively, providing dynamic binding information. In our experiments, the affinity of BIBF for VPS18 protein was determined using different concentration conditions (Figure 4C). Second, the VPS18 coupled to the metal surface was analyzed to see whether it binds to BIBF and produces different resonance angles by monitoring the changes in resonance angles. The experimental results showed that the binding rate constant (kon) was 9.885 × 10^2^ M^−1^s^−1^, and the dissociation rate constant (koff) was 1.118 × 10^−2^ s^−1^. The data were analyzed using the Biacore T200 analysis software (Kinetics Analysis Version 2.0.0.3) and 1:1 binding model whose calculated equilibrium dissociation constant (KD) was 11.31 μM (Figure 4D). Experimental confirmation of the molecular docking predictions led to the same conclusion.

To further investigate the role of VPS18 in the mechanism of BIBF action, we first knocked down the expression level of VPS18 in U251 and U87 cells using VPS18-siRNA. The knockdown efficiency was verified by qPCR (Figure 4E) and Western blotting (WB) techniques (Figure 4F), and the results demonstrated that VPS18 expression was significantly suppressed in both cell lines. After successfully knocking down VPS18, we examined the effect of BIBF on the half-inhibitory concentration (IC50) of U251 and U87 cells using the CCK-8 assay. The experimental results indicated that the IC50 curve of BIBF shifted significantly to the right after transfection with VPS18-siRNA (Figure 4G,H), suggesting that the cells were less sensitive to BIBF. Additionally, we assessed apoptosis in U251 and U87 cells using flow cytometry and found that BIBF-induced apoptosis was markedly reduced following the transfection with VPS18-siRNA (Figure 4I,J). These findings suggest that VPS18 knockdown may impair BIBF’s ability to promote apoptosis in glioblastoma cells. To further verify the impact of BIBF on autophagy in U251 and U87 cells, we analyzed the expression of key autophagy-related proteins by Western blotting. The results revealed that BIBF significantly inhibited autophagy in both cell lines; however, this autophagy inhibitory effect was notably reversed when VPS18 was knocked down (Figure 4K). This outcome indicates that VPS18 plays a crucial role in the regulation of autophagy in U251 and U87 cells in response to BIBF treatment. In summary, our experimental results indicate that the effects of BIBF on glioblastoma cells are primarily mediated through VPS18. On one hand, BIBF enhances apoptosis by inhibiting VPS18-regulated autophagy; on the other hand, the knockdown of VPS18 reverses the autophagy inhibitory effect of BIBF, thereby attenuating its pro-apoptotic effects. As a key regulator in the autophagy pathway, VPS18 knockdown’s reversal of BIBF efficacy highlights the importance of VPS18 in modulating the biological behavior of tumors in U251 and U87 cells. BIBF demonstrates a dual effect on these cells through the autophagy and apoptosis pathways regulated by VPS18. Furthermore, the expression level of VPS18 may be closely related to the malignancy and drug sensitivity of glioblastoma. By harnessing the biological characteristics of VPS18, synergistic drugs could potentially be developed in combination with BIBF in the future, thereby enhancing therapeutic effects on glioblastoma, particularly through targeting the autophagy pathway, which is especially crucial.

### 2.5. PLGA-Polymrized BIBF NPs Solve the Problem of BIBF’s Inability to Cross the Blood–Brain Barrier

**Figure 5:**
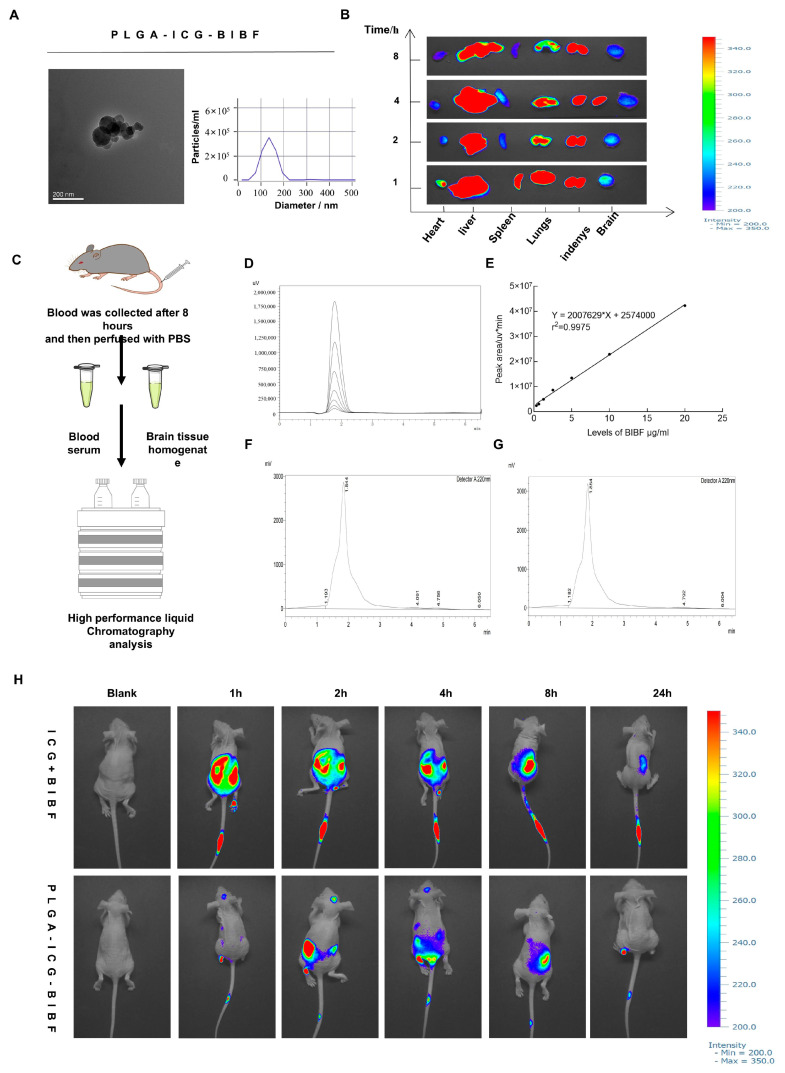
PLGA-BIBF NPs target GBM across the blood–brain barrier. (**A**) Transmission electron microscopy observation of the morphology of PLGA-polymerized BIBF NPs. (**B**) Collection of ICG-labeled PLGA-BIBF at different time points, isolation of in vivo organs, and in vitro acquisition of near-infrared fluorescence images of heart, liver, spleen, lungs, and brain. (**C**) Verification of the pattern maps of PLGA-BIBF through the blood–brain barrier. (**D**) Peak out at 220 nm for different concentrations of BIBF. (**E**) Standard curve of BIBF at 220 nm. (**F**) Analysis of PLGA-BIBF in serum, (**G**) Analysis of PLGA-BIBF in intracranial, serum, and brain tissue homogenate with the same result of peak-out of PLGA-BIBF at 220 nm at 1.5 min. (**H**) Acquisition of near-infrared fluorescence images of ICG-labeled PLGA-BIBF, BIBF intracranial glioblastoma at different time points. ICG-labeled PLGA-BIBF was acquired at different time points, the organs were separated in vivo, and the NIR fluorescence images of the heart, liver, spleen, lungs, and brain were obtained in vitro.

PLGA has been shown to have good biodegradability, controlled release properties, and low toxicity in drug delivery studies, and it can modify water-soluble and fat-soluble drugs to prolong the duration of drug action and also improve the stability and bioavailability of drugs [36]. Considering the difficulty that BIBF itself cannot cross the BBB, we used PLGA to encapsulate BIBF to improve its targeting ability. Then, to verify the morphology and particle size distribution of PLGA-encapsulated BIBF NPs (PLGA-BIBF), we used transmission electron microscopy (TEM) for observation. The results showed that the purified PLGA-BIBF NPs were uniformly spherical with a particle size distribution between 20 and 200 nm (Figure 5A), which is consistent with the particle size range of nanodrugs. To further investigate the distribution of PLGA-BIBF in different organs in vivo, we established an intracranial xenograft tumor model in BABL/C nude mice. ICG-labeled PLGA-BIBF was injected through the tail vein, and near-infrared fluorescence images were acquired at different time points (Figure 5B). The imaging results showed that the blood flow through the carotid and cerebral arteries carried a large amount of PLGA-BIBF, which may have led to blood flow interference in the observed fluorescence signals in the brain region. To exclude blood flow interference, we further removed the major organs and brains of nude mice for in vitro imaging analysis. The results clearly showed that ICG-labeled PLGA-BIBF was significantly distributed in the brain, thus excluding the interference of blood flow signals. This indicated that PLGA successfully delivered BIBF to the brain.

To further verify whether PLGA-BIBF can cross the blood–brain barrier at the level of quantitative biodistribution analysis, we designed (Figure 5C) an experimental protocol in which we injected PLGA-BIBF through the tail vein, anesthetized and killed the mice 8 h after administration, collected venous blood samples, separated the brain and brain region samples, and used high-performance liquid chromatography (HPLC) to analyze the blood and different brain regions for the BIBF content in the blood and different brain regions were analyzed by HPLC. The standard curves of PLGA-BIBF (Figure 5D,E) showed that its intracranial content (Figure 5G) could be accurately quantified by the peak time at 220 nm at 1.5 min, which was consistent with the peak time of BIBF in serum (Figure 5F). This result indicated that PLGA-BIBF successfully crossed the blood–brain barrier and entered the brain region. Next, to investigate the targeting ability of PLGA-loaded BIBF against intracranial glioblastoma in vivo, we acquired near-infrared fluorescence images at different time points after injection of ICG-labeled PLGA-BIBF through the tail vein in an in situ xenograft tumor nude mouse model (Figure 5H). The results showed that PLGA-BIBF significantly aggregated in the brain within 1 h–8 h after drug injection, and the fluorescence intensity peaked at about 2 h after injection and then gradually decreased with time, and the fluorescence signal completely disappeared at 24 h after injection. This suggests that PLGA-BIBF can rapidly reach the brain after injection and gradually metabolize after aggregation in the tumor region. The results of this study demonstrate that PLGA-BIBF NPs not only have nanomorphology and particle size distribution but also can successfully cross the blood–brain barrier and target the intracranial glioblastoma site, and PLGA-BIBF NPs provide a potential new strategy for the precise treatment of glioblastoma.

### 2.6. PLGA-Delivered Nintedanib Inhibits GBM Growth to Improve Survival in BALB/c Nude Mice

**Figure 6:**
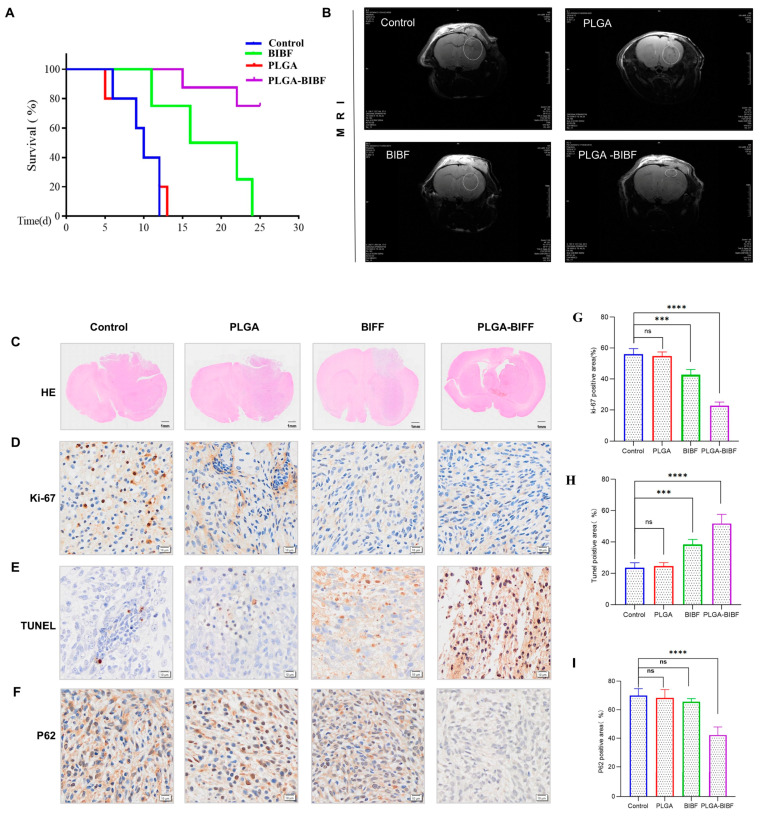
PLGA-BIBF significantly inhibited GBM growth in vivo. (**A**) Survival curves for intracranial xenograft tumor models in BALB/C nude mice. (**B**,**C**) Representative images of MRI and HE staining from nude mice with in situ implanted tumors after treatment with PBS, PLGA, BIBF, and PLGA-BIBF (6 mice per group). (**D**–**F**) Detection of Ki67, TUNEL assay, and P62 expression via immunohistochemistry. (**G**) Quantification of Ki67-positive cells across different treatment groups. (**H**) Quantification of the apoptosis rate in various treatment groups. (**I**) Quantification of P62-positive cells in different treatment groups. Comparisons with the control group are as follows: ns, no significant difference compared to control, *** *p* <0.001, compared to control **** *p* < 0.0001, compared to control.

To further validate the therapeutic effect of PLGA-BIBF on the intracranial xenograft tumor model in BABL/C nude mice, we started the treatment on the 10th day after tumor implantation and divided the nude mice into four groups, namely PBS, PLGA, BIBF, and PLGA-BIBF, and regularly injected the corresponding drugs on a daily basis. The results of tumor volume measured by MRI (Figure 6B) showed that compared with the control group, injection of PLGA or BIBF alone did not significantly inhibit tumor growth, while the PLGA-BIBF treatment group significantly inhibited tumor growth. The results of H&E staining (Figure 6C) further supported the conclusion that neither PLGA nor BIBF groups completely inhibited tumor growth, whereas the PLGA-BIBF group significantly slowed tumor growth, although it did not completely eradicate the tumor.

To further investigate the anti-tumor mechanism of PLGA-BIBF, we detected the expression levels of Ki67, P62, and TUNEL in the transplanted tumors by immunohistochemical analysis. The results showed that the level of Ki67 (proliferation marker) was significantly decreased, while the level of TUNEL (apoptosis marker) was significantly increased in the PLGA-BIBF treatment group (Figure 6D,E). Meanwhile, there was no significant change in the expression level of P62 (autophagy marker) in the BIBF and PLGA groups compared with the control group, while the level of P62 was significantly decreased in the PLGA-BIBF group (Figure 6F). These results indicated that PLGA-BIBF inhibited autophagy in glioblastoma and significantly induced apoptosis in tumor cells, thereby inhibiting intracranial tumor growth. In conclusion, this study demonstrated that PLGA-BIBF has good anti-glioblastoma effects, which improved the quality of survival in nude mice by significantly inhibiting tumor growth, inducing apoptosis of tumor cells, and suppressing autophagy, demonstrating its potential as a therapeutic agent for GBM.

## 3. Discussion

BIBF is a multi-targeted tyrosine kinase inhibitor currently used to treat a variety of diseases [37], including idiopathic pulmonary fibrosis, systemic sclerosis-associated interstitial lung disease, and lung cancer. In studies of cancers such as breast cancer [38], bladder cancer [39], and non-pancreatic neuroendocrine tumors (NETs) [40], BIBF has been shown to inhibit tumor cell proliferation while significantly reducing tumor angiogenesis. Additionally, BIBF has been implicated in the pathophysiology of the central nervous system. However, due to the blood–brain barrier (BBB), many anticancer drugs cannot effectively penetrate the central nervous system [37], which poses a major obstacle in the treatment of glioblastoma multiforme (GBM). In this study, we found that BIBF induced apoptosis in U251 and U87 cells and, for the first time, proposed its mechanism of action as a potential autophagy inhibitor. Given that BIBF itself struggles to cross the blood–brain barrier, we encapsulated it using NPs to overcome this limitation and successfully delivered BIBF to the central nervous system. The nanodelivery system significantly enhanced the distribution efficiency of BIBF in intracranial space and effectively inhibited the growth of GBM in a mouse model. This finding opens a new research direction for the application of BIBF in the treatment of CNS malignancies.

Our initial findings revealed that BIBF could effectively inhibit the proliferation of U251 and U87 cells through a small-molecule drug library screen, suggesting a potential therapeutic strategy for anti-tumor therapy with biological responsiveness [41]. GBM is a highly aggressive malignant brain tumor, and to further investigate its invasion-related mechanisms, we conducted a detailed examination of BIBF in a controlled in vitro environment at the molecular and cellular levels. In comparison to the control group, BIBF treatment demonstrated a notable reduction in the viability of U251 and U87 cells, accompanied by a marked inhibition of their proliferation, migration, and invasion abilities. These mechanisms represent pivotal factors contributing to the high recurrence rate and poor prognosis associated with GBM. Of particular interest was the observation that BIBF induced apoptosis in U251 and U87 cells while simultaneously inhibiting their autophagic processes. The role of autophagy in tumor cells is dual, either promoting tumor progression by maintaining cell survival or possibly inhibiting tumor growth under certain conditions. Notably, our findings are consistent with those of Hegedüs et al. in their colon cancer study [12], in which they found that BIBF could induce apoptosis in colon cancer cells by activating protective autophagy. Based on these findings, we further validated the mechanism of action of BIBF in GBM therapy, which is to induce apoptosis by inhibiting autophagy. The in vitro experimental results collectively demonstrate that BIBF exerts a pronounced inhibitory effect on U251 and U87 cells, providing substantial evidence in support of its potential as an anti-GBM therapy. Furthermore, to enhance the efficacy and targeting of BIBF, we encapsulated it in PLGA NPs to achieve more efficient delivery through the use of enhanced permeability and retention (EPR) [42]. In the field of cancer therapy, the EPR effect has emerged as a pivotal area of investigation in drug delivery research, largely due to its distinctive tumor-targeting mechanism. By employing this strategy, we were able to successfully deliver BIBF to the intracranial GBM target area in mice, resulting in notable inhibition of GBM progression.

Herhaus et al. demonstrated that autophagy is a meticulously regulated process [43], primarily functioning to degrade non-functional cellular components via lysosomes, maintain energy balance during starvation, and eliminate noxious substances. A pivotal step in autophagy is the formation of phagocytic vesicles, which encapsulate intracellular “cargo” to form an autophagosome [44]. The subsequent fusion of the autophagosome with lysosomes completes the degradation process. The formation of phagocytic vesicles is precisely controlled by multiprotein complexes, while the fusion of autophagosomes with lysosomes and the segregation of cargoes during selective autophagy, directly determine the specificity of the degradation process, a mechanism that plays an important role in tumor progression [25]. In the present study, we found that BIBF has a significant effect on the autophagy–lysosome system in GBM cells. To elucidate the underlying molecular mechanism, we conducted an analysis of the interaction between BIBF and autophagy-related proteins utilizing molecular docking methods. Molecular docking serves as a predictive tool that employs the three-dimensional structures of compounds and proteins to “fit” compounds to target proteins, thereby facilitating the prediction of their binding modes and binding energies. This approach holds significant value in drug discovery and biological research. The results of our study demonstrate that BIBF exhibits substantial binding affinity to vps18, a pivotal protein within the autophagy pathway. The binding energy of BIBF to vps18 was further validated by plasmon resonance experiments, and the results corroborated the predictions made by molecular docking. These findings suggest that BIBF may be involved in the pathological progression of GBM by affecting cargo segregation and degradation, thereby interfering with the autophagic process.

VPS18 plays a pivotal role in lysosomal maturation as a core component of the vesicular protein sorting (VPS-C) core complex [45]. VPS proteins comprise homotypic fusion and vesicular protein sorting-binding complexes, which include VPS11, VPS16, VPS18, VPS33A, VPS39, and VPS41 [46]. The HOPS-specific subunit VPS41 has been reported to enhance the viability of dopaminergic neurons in Parkinson’s disease [47], support the involvement of VPS41 dysfunction in the disease using a zebrafish model, suggest lysosomal dysregulation throughout the brain, and indicate cerebellar and microglial cell abnormalities in the presence of VPS41 mutations. This underscores the importance of HOPS complex activity for brain function. Concurrent studies demonstrate that autophagy involves a critical step in substrate degradation: the fusion of lysosomes. Furthermore, Zhang et al. identified the C9orf72-RAB39A-HOPS axis as a key regulatory element in the model of autophagosome–lysosome fusion [48]. Numerous studies have reported elevated VPS18 expression in various types of cancers [49], including prostate, bladder, and lung cancers, compared to adjacent non-cancerous tissues. This heightened expression is associated with a poor prognosis in these malignancies [50]. In this study, we investigated the impact of VPS18 on the efficacy of BIBF in targeting glioblastoma (GBM) by transfecting VPS18-siRNA into U251 and U87 cells. Our experimental findings demonstrated that the targeting capacity of BIBF was significantly diminished in VPS18-silenced cells. Specifically, we found that the anti-GBM efficacy of BIBF was enhanced at equivalent concentrations, while apoptosis levels were reduced, and autophagy inhibition was significantly improved. These results suggest that BIBF’s effectiveness is contingent on its interaction with VPS18. Further studies revealed that BIBF affects autophagosome–lysosome fusion by forming a π-bond with VPS18, which induces apoptosis and inhibits autophagy. This study provides the first evidence for the mechanism through which BIBF exerts its antitumor effects by targeting VPS18.

The BBB is a specialized vascular structure that plays a critical role in protecting and nourishing neurons and glial cells in the brain while impeding the delivery of drugs to the central nervous system [51]. This barrier is of significant interest in studies on brain function and the development of treatment strategies for brain tumors [52]. However, in the treatment of glioblastoma (GBM), the presence of the BBB significantly hinders the effective uptake of anticancer drugs, thereby accelerating the spread and metastasis of GBM [53]. Consequently, the development of nano-loaded drug delivery technology has emerged as an innovative therapeutic strategy capable of overcoming the BBB and delivering drugs to the central nervous system in a safe and efficient manner [54]. Among various drug delivery methods, PLGA NPs (NPs) have emerged as a particularly promising solution. These NPs, composed of long-chain molecules linked by covalent bonds, offer unique advantages for facilitating drug delivery [55]. For instance, PLGA NPs have been shown to enhance the uptake of methotrexate by tumor cells in a transferrin-mediated manner [56]. Additionally, poly(alkyl cyanoacrylate) has demonstrated the capacity to penetrate the membranes of tumor cells that overexpress P-glycoprotein, which plays a role in multidrug resistance [57]. The advantages of PLGA include its good biocompatibility and biosafety, as well as its flexible modulation properties and ease of surface modification (e.g., conjugation with antibodies or peptides). Furthermore, PLGA NPs can be designed to be sensitive to various stimuli (e.g., pH, reactive oxygen species (ROS), redox state, temperature, or light), further enhancing their effectiveness in targeted drug delivery [58]. The in vivo experimental results of this study demonstrated that PLGA NPs successfully delivered nintedanib (BIBF) to the tumor region in the brain and exerted significant anti-tumor effects. Recent research has revealed advancements in optimizing the pharmacokinetics and biodistribution of polymeric NPs, including benefits such as prolonged circulation time, prevention of macrophage clearance, increased drug retention in tissues, and reduced toxicity. This study further demonstrated that PLGA-delivered nintedanib could cross the blood–brain barrier and effectively inhibit the growth of glioblastoma, providing strong support for the development of a safe and efficient therapeutic strategy for glioblastoma.

Notwithstanding the significant findings of this study, certain limitations must be acknowledged. For instance, an in-depth investigation of the molecular conformation of VPS18 has yet to be conducted, which hinders our ability to clarify the specific site of action of nintedanib on VPS18. To further explore the molecular mechanism by which nintedanib regulates VPS18, we plan to conduct point mutation or multi-point mutation studies on key amino acid sites of VPS18 in glioblastoma models. These studies will elucidate the molecular basis of its interaction with BIBF and provide a more detailed theoretical foundation for the clinical trials of nintedanib.

## 4. Materials and Methods

### 4.1. Drug Preparation and Reagents

In this study, we utilized several reagents and antibodies, including Nintedanib (Targetmol, MA, USA, 65624-17-5), Z-VAD-FMK (Targetmol, MA, USA, 187389-52-2), bafilomycin (Targetmol, MA, USA, 88899-56-3), Cell Counting Kit-8 (CCK-8) (Sangon Biotech, Suzhou, China, C6030), PE Annexin V (Yeasen, Shanghai, China, 40,302), and a lysosomal red fluorescent probe (Beyotime, Shanghai, China, C1047S). The following primary antibodies were obtained from Cell Signaling Technology (CST): anti-Bax (CST, Beverly, MA, USA, #2772), anti-Bcl-2 (CST, MA, USA, #3498), anti-cleaved caspase 3 (CST, MA, USA, #D175), anti-Beclin-1 (CST, MA, USA, #4122S), anti-cathepsin D (CST, MA, USA, #2284), anti-caspase 3 (CST, MA, USA, #3218), anti-LC3 (CST, MA, USA, #3868), anti-P62 (CST, MA, USA,#5114S). The anti-β-actin (Abcam, Cambridge, UK, #ab8226) antibodie was obtained from Abcam. Additionally, The following primary antibodies were obtained from Proteintech: anti-Lamp1 (Proteintech, Wuhan, China, #67300-1-Ig), anti-Lamp2 (Proteintech, Wuhan, China, #66301-1-Ig), anti-Rab7 (Proteintech, Wuhan, China, #55469-1-AP), and anti-Vps18 (Proteintech, Wuhan, China, #10901-1-AP). siRNA Vps18 (Hanbio, Shanghai, China,) CM5 microarray (CBG, London, UK).

### 4.2. Cells and Culture Conditions

U251 and U87 MG were purchased from the American Type Culture Collection cell bank and were maintained at the Key Laboratory of Neurosurgery, Second Hospital of Lanzhou University. DMEM complete medium containing 10% fetal bovine serum, 100 U/mL penicillin, and 100 μg/mL streptomycin was used to resuscitate the cells. Culture conditions were set as follows: 37 °C, 5% CO_2_, and constant humidity.

### 4.3. Small-Molecule Drug Library Screening

Digest the logarithmic growth phase glioblastoma cells with trypsin, count the cells, and then seed them into a 96-well plate for incubation in a controlled environment. Dilute the molecular library drugs according to the instructions provided. Replace the original medium containing the drugs in the 96-well plate with the CCK-8 reagent as per the protocol. Incubate the cells in the dark at 37 °C for 2 h and then measure the absorbance at 450 nm using a Bio-Tek enzyme labeling instrument. Calculate cell viability according to the CCK-8 reagent instructions.

### 4.4. Cell Viability and Cytotoxicity Assays

Glioblastoma cells in the logarithmic growth phase were counted and seeded into 96-well plates and incubated in a warm box for 24 h. The molecular library drug was diluted according to the instructions. The original drug-containing medium was renewed in the 96-well plate by adding CCK8 liquid according to the instructions in the user manual. The cells were then incubated at 37 °C for 1.5 h in the dark, and the absorbance at 450 nm was measured using a Tio-Tek Enzyme Labeler. Cell activity analysis data were calculated according to the instructions

### 4.5. Cell Cycle Assay

Glioblastoma cells were inoculated into 6-well plates and monitored under a microscope until they fully adhered. The medium was then replaced with serum-free DMEM, and the cells were cultured for an additional 8 h before being treated with BIBF for 48 h. Following treatment, the cells were harvested by trypsin digestion and washed three times with PBS. A total of 1 mL of DNA staining solution and 10 µL of permeabilization solution were added to the cells. The mixture was gently vortexed and incubated in the dark at room temperature according to the manufacturer’s instructions. Prior to analysis, the samples were filtered through a 200-mesh sieve, and DNA content was assessed using flow cytometry. The results were visualized and analyzed using FlowJo (version 10).

### 4.6. Monolayer Wound-Healing and Transwell Assays

The following procedure was followed: First, mark horizontal lines on the underside of a 6-well plate and then seed glioblastoma cells into the wells. Incubate in a CO_2_ incubator and observe under the microscope until the cells reach approximately 70% confluence. Replace the medium with serum-free DMEM and continue incubation for an additional 12 h. In a sterile handling area, use the tip of a 10 μL pipette to scratch the surface of the cells and then treat the cells with different concentrations of nintedanib. Capture images at various time points using an inverted phase contrast microscope. Perform the procedure on ice within a sterile environment. First, layer 50 μL of the appropriate concentration of matrix gel to cover the chamber and then incubate in a CO_2_ incubator to allow it to solidify. Next, seed glioblastoma cells into the chamber and add the corresponding concentrations of the drug to the lower chamber. After 48 h, gently remove the matrix gel using a cotton swab, fix the cells, stain them, and capture images using a microscope.

### 4.7. Apoptosis Assay

The following procedure was followed: Treat glioblastoma cells with DMSO, BIBF, and H_2_O_2_ for 48 h. Wash the cells twice with cold PBS using a non-EDTA enzyme solution. Resuspend the cells in 100 µL of binding buffer. Next, add 5 µL of V-Alexa and 5 µL of propidium iodide (PI) and incubate in the dark according to the manufacturer’s instructions. Before analysis, adjust the cell density in each sample with binding buffer and filter through a 200-mesh sieve to prevent clogging. Perform flow cytometry analysis within one hour. Visualize the flow cytometry results using FlowJo (10.8.1) software.

### 4.8. Transmission Electron Microscopy

The following procedure was followed: Treat U251 cells with DMSO and BIBF for 48 h. Digest the cells with trypsin, centrifuge to collect them, and then wash them several times with PBS to resuspend. Fix the cells with a fixation solution for electron microscopy. Next, incubate them in 1% osmium tetroxide. After dehydration, stain with uranyl acetate and observe the cellular microstructure using a transmission electron microscope (JEM-1230, JEOL Ltd., Tokyo, Japan).

### 4.9. Plasmid Infection

The following procedure was followed: Transfect dual fluorescent plasmid GFP-RFP-LC3 into glioblastoma cells. After 24 h of transfection, observe the fluorescence signals using a fluorescence microscope to confirm the expression of the fluorescent proteins. Subsequently, we treated the tumor cells with drugs, and after 24 h of treatment, we collected fluorescence images of individual cells using the LSM-9000 microscope.

### 4.10. Western Blot Analysis

Cells were treated with RIPA lysis buffer containing a combination of protease and phosphatase inhibitors, and proteins were extracted from cells and tissues by low-temperature ultrasonic wall disruption. Proteins were quantified using the BCA kit assay prior to the experiment, and samples were stored at −20 °C prior to Western blotting analysis. After Western blotting analysis, bands were exposed, and images were analyzed for the optical density of the bands using ImageJ (2024) software.

### 4.11. Molecular Docking

The 2D structure of BIBF was created using Chem Draw (2018) software and then imported into Chem3D. Energy minimization was performed using the MM2 module to obtain the most stable conformation, and the resulting file was saved in mol2 format. The docked protein structures were obtained from the PDB database and visualized using PyMOL. The structures were then prepared using MGLTools 1.5.6 with the process of merging non-polar hydrogens, adding hydrogen atoms, removing water molecules, and calculating charges, with BIBF as the ligand and autophagy-associated proteins as the receptor, and saved in pdbqt format. Docking was performed using AutoDock Vina 1.1.2, and the conformations with the highest binding affinity scores were selected for further visualization in PyMOL and Discovery Studio.

### 4.12. Protein and Small Molecule Interaction Analysis

A BI-4500 instrument was used to analyze the interaction between VPS18 and BIBF to detect the magnitude of affinity. The CM5 chip was installed on the prism holder, and degassing treatment was performed. The temperature and flow rate were set. Anhydrous ethanol was used for sensor calibration, and the CM5 chip was activated. The protein was immobilized on the surface of the CM5 chip, and the unbound sites of the chip were blocked with ethanolamine. Next, 10× PBS was used as a blank control, and small molecules were injected from low to high concentrations. The test results were analyzed with BI data evaluation software (Kinetics Analysis Version 2.0.0.3).

### 4.13. PLGA for Dintedanib Delivery

To prepare the polyvinyl alcohol (PVA) solution, 0.5% PVA was heated overnight in an oven set to 70 °C. The PLGA:PVP:BIBF mixture (in a ratio of 20:1:2) was then gradually dissolved in the 0.5% PVA solution mixed with ClO_2_ (at a ratio of 4:1). This mixture was placed in an ice bath for 5 min and stirred at room temperature at 200 rpm for 24 h. Afterward, the mixture was centrifuged at room temperature to eliminate larger vesicles. The resulting precipitate was washed multiple times and then freeze-dried for 24 h. The synthesized drug was examined using an electron microscope.

### 4.14. High-Performance Liquid Chromatography Analysis

This study utilized a high-performance liquid chromatography (HPLC) system manufactured by Agilent Technologies, located at the Cuiying Platform. The primary equipment included the Agilent G1315D pump and the Agilent G1311 detector. The chromatographic column used was a C18 type, with dimensions of 250 × 4.6 mm and a particle size of 5 μm. The mobile phase consisted of a methanol/water mixture containing 0.05% triethylamine, adjusted to a pH of 5.0 using phosphoric acid, in a ratio of 90:10. During the experiments, the temperature of the chromatographic column was maintained at 30 °C to ensure stable separation efficiency. The detection wavelength was set to 220 nm to enhance the sensitivity of the analyte detection. For each analysis, a standard curve was employed for quantitative analysis, achieving a correlation coefficient (R^2^) of 0.9975, indicating a strong linear relationship. The injection volume was fixed at 10 microliters to ensure the accuracy and repeatability of the analytical results.

### 4.15. Animals Studies

The experimental qualifications were obtained with the approval of the Animal Ethics Committee of the Second Hospital of Lanzhou University. Our team established an in vivo intracranial tumor model in a specific pathogen-free (SPF) laboratory by using a stereotactic approach to slowly inject a total of 1.5 µL of U87 cell suspension into the brains of mice at a rate of 1 µL/min. The injection site was positioned 1 mm posterior to the fontanelle, 2 mm lateral to the median suture, and at a depth of 3.5 mm. Tumor formation was assessed via MRI ten days post-implantation. Subsequently, we administered the corresponding drugs intraperitoneally according to the assigned groups for seven consecutive days and conducted ongoing MRI (uMR 9.4T, United Imaging Life Science Instrument, Wuhan, China) imaging analysis. The mice were euthanized on Day 25, and their brain tissues were collected for further research.

### 4.16. In Vivo Imaging of Small Animals

To visualize the drug dynamics, nude mice received ICG-labeled PLGA-BIBF via tail vein injection after ten days of tumor growth. Following injection, the mice were anesthetized using isoflurane in an anesthesia box for five minutes. They were then placed into a small animal in vivo imaging system for the detection of the fluorescent drug. The imaging system was configured with an excitation wavelength of 780 nm, an emission wavelength of 830 nm, and an exposure time of 900 milliseconds. Live imaging of the intracranial xenograft tumors was conducted before injection and at 1 h, 4 h, 8 h, and 24 h post-injection to measure fluorescence intensity and assess whether the drug had crossed the blood–brain barrier.

### 4.17. Immunohistochemical and Immunofluorescence Assessment

After 25 days following tumor implantation, all animals were euthanized using chloral hydrate. The first step involved perfusion with PBS, followed by perfusion with 4% paraformaldehyde. The brains were then carefully separated, fixed in 4% paraformaldehyde, and stored at a low temperature (4 °C). After 7 days, the samples were dehydrated and embedded in paraffin. Paraffin sections, measuring 3 μm to 5 μm in thickness, were stained using hematoxylin and eosin (H&E) and corresponding immunohistochemical staining methods to identify tumor regions and assess the proliferation, autophagy, and apoptosis rates of tumor cells.

### 4.18. Statistical Analyses

We analyzed all experimental data using GraphPad Prism (10.4.0.612) software. To compare the data, we employed Tukey’s multiple comparison test, establishing a significance threshold of *p* < 0.05. The results are presented as the mean ± standard deviation. A *p*-value of less than 0.05 is considered statistically significant. This rigorous statistical approach ensures that our findings are robust and reliable, providing confidence in the interpretations drawn from the data.

## 5. Conclusions

**Figure 7:**
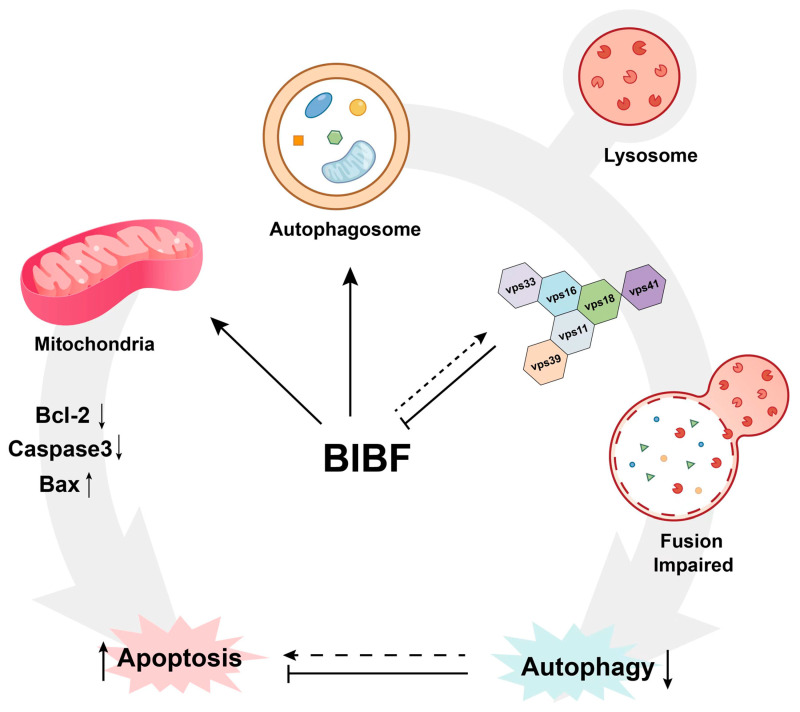
The schematic of the proposed mechanism illustrates how the interaction between BIBF and VPS18 affects the binding of lysosomes and autophagosomes, inhibiting autophagy and enhancing cell apoptosis.

In conclusion, the present study demonstrated that nintedanib is a novel autophagy inhibitor that inhibits autophagy and further promotes apoptosis in GBM cells by affecting lysosome-autophagosome fusion. The present data also showed that in vivo delivery of nintedanib via PLGA inhibits the growth of GBM xenografts through the blood-brain barrier (Figure 7). Thus, nintedanib has great research value and potential as an autophagy flux inhibitor in anticancer therapy.

## Data Availability

The data presented in this study are available on request from the corresponding author. The data are not publicly available due to subsequent research is still ongoing.
